# Oral respiration modulates sensory and cognitive brain potentials differently than nasal respiration

**DOI:** 10.1038/s41598-025-12518-1

**Published:** 2025-08-11

**Authors:** Viviana Leupin, Juliane Britz

**Affiliations:** Department of Psychology, University of Fribourg, University of Fribourg, Rue P.-A. Faucigny 2, Fribourg, CH-1700 Switzerland

**Keywords:** Consciousness, Brain–body interaction, EEG, Respiratory phase, Oral breathing, Consciousness, Perception, Neuroscience, Cognitive neuroscience

## Abstract

Respiration influences brain activity and awareness through two mechanisms: direct rhythmic entrainment via olfactory bulb (OB) stimulation and indirect modulation through baroreceptor (BR) activity fluctuations that affect cortical excitability. During nasal breathing, both mechanisms are active and can influence sensory event-related potentials (ERPs). To isolate the contribution of BR activity, we tested the same paradigm during oral breathing, which eliminates OB stimulation. Results showed that sensory ERPs varied with the cardiac (with delayed timing) but not with the respiratory phase. The P3a component was modulated by awareness only when BR activity was low (during inhalation and diastole), while the visual awareness negativity (VAN) and P3b were modulated by awareness across all conditions. These findings indicate that BR fluctuations alone cannot fully account for the respiratory modulation of awareness-related ERPs, but they require entrainment of brain activity modulated by mechanical OB stimulation.

**Supplementary Information:**

The online version contains supplementary material available at 10.1038/s41598-025-12518-1.

## Introduction

Certain conditions allow researchers to dissociate perception from sensation, such that identical physical stimuli give rise to different perceptual outcomes. Stimuli presented at the sensory threshold are equally likely to be perceived or missed, with awareness fluctuating apparently randomly from trial to trial, which makes them powerful tools for studying the neural correlates of consciousness. Since trial-by-trial fluctuations in awareness cannot arise from the stimulus itself, they reflect either differences in the responses of the brain to the stimulus or fluctuations in the pre-stimulus brain state. Both stimulus-evoked and state-dependent differences in brain activity make complementary contributions to our understanding of how conscious awareness arises.

 Contrasting event-related potentials (ERPs) evoked by a stimulus when it is perceived or missed can elucidate the time-course underlying the emergence of awareness. However, it remains debated whether sensory processing (reflected by the P1 component, ~ 80–120 ms after stimulus onset) differs between aware and unaware perception^1,2^ or not^3–5^. Both perceptual and post-perceptual processes reflected by the mid-latency Visual Awareness Negativity (VAN) and the P3b/LPC component are reliably modulated by awareness and more pronounced for aware vs. unaware perception^5–8^, suggesting that awareness is only resolved after the initial sensory processing. Whether the VAN^9–11^ or the P3b/LPC^5–7^ is the earliest marker of awareness remains a matter of debate.

Apparently random fluctuations in awareness have been reliably attributed to trial-by-trial variations in spontaneous brain activity. Both local and global EEG measures can predict whether an upcoming visual stimulus is perceived or missed. Local pre-stimulus alpha power over occipital areas, an indicator of cortical excitability, is lower for perceived than for missed stimuli^12–14^, and global pre-stimulus microstates can likewise predict differences in awareness^15–17^.

The brain continuously integrates signals from both outside and inside the body, and awareness fluctuates not only with the state of the brain but also with cyclic fluctuations in bodily rhythms. The cardiac rhythm is the first pacemaker of the organism^18^and its cycle is subdivided into the systolic (contraction / ejection of blood) and diastolic (relaxation / influx of blood) phases. Visual^19–22^, auditory^23^ and somatosensory^24,25^ stimuli are less likely detected and are identified more slowly during systole. These effects are likely generated by the rise in activity of stretch sensors (baroreceptors (BRs)) located in the aortic arch and the carotid sinus during the systole that relay information about the increased blood pressure to the brain. The processing of this interoceptive afferent visceral signal decreases cortical excitability^26^which can be described in the framework of gain control as the slope of the input and output (I/O) function. When BR activity is low, the gain function is steeper, which amplifies relevant (sensory) stimuli and attenuates irrelevant (interoceptive) signals. Conversely, increased BR activity decreases the gain and so reduces the sensitivity of the I/O function and thus the ability of the brain to distinguish between irrelevant interoceptive signals and relevant exteroceptive stimuli^27^.

BR activity also fluctuates cyclically with the phase of respiration, which is another fundamental bodily rhythm. It is essential for gas exchange (O_2_ for CO_2_) but can also modulate perceptual awareness. Subjects perform better in a visuo-spatial task and detect visual stimuli more easily during inhalation^28,29^. Moreover, visual stimuli presented during inhalation elicit larger ERPs^29^and subjects synchronize their breathing with task demands^29,30^. However, alternative accounts have reported improved detection rates during exhalation in both visual^31^ and somatosensory^30^ modalities. We have recently shown that awareness-related ERPs for visual threshold stimuli are modulated similarly by both the cardiac and respiratory phase, with cyclic fluctuations of BR activity mediating gain control that affect both the sensory processing and the trajectory of brain activity when subjects became aware of a threshold stimulus^32^. Taken together, these findings suggest that multiple physiological mechanisms may link respiration and perceptual awareness.

Respiration directly drives both broad-band resting-state^28^ and task-related oscillatory brain activity, both in rodents^33–36^ and humans^37,38^: local field potential (LFP) amplitudes and power in structures of the limbic system crucial for memory consolidation are coupled to the respiratory cycle^38^ and vary with the respiratory frequency of the organism^34,35,39^. Moreover, mechanical stimulation of the olfactory bulb (OB) during respiration drives phase-amplitude coupling (PAC, amplitude increase in a faster frequency band coupled to the phase of a slower frequency band) in these regions between the theta phase and gamma amplitude in both humans^38^ and in rodents^34^. Oral breathing^38^ and OB ablation^34,35^which bypass mechanical OB stimulation abolish the coupling between gamma amplitude and theta phase, suggesting that respiration acts as an oscillatory scaffold underlying the coordination of activity between brain areas^35^.

Respiration can affect awareness also indirectly via cardio-respiratory coupling known as respiratory sinus arrhythmia (RSA), which accelerates the heart rate during inhalation and decelerates it during exhalation^40^. RSA reflects the adaptive coordination between the sympathetic and parasympathetic systems^41^ and is mediated by BR activity: during exhalation, both BR activity and vagal (parasympathetic) output increase, which triggers the baroreflex that decelerates the heart rate; during inhalation, low BR activation and higher sympathetic tone accelerate the heart rate^40^.

Critically, direct and indirect mechanisms are confounded during nasal breathing, which involves both OB stimulation and RSA. It remains thus unclear whether the modulation of awareness-related neural responses by respiration can be solely driven by BR activity mediated gain control or whether that requires OB mediated neural synchronization. To disentangle the contribution of these two mechanisms, we examined awareness-related brain responses during oral breathing, which prevents mechanical OB stimulation while preserving BR activity mediated RSA and gain control. This approach allowed us to isolate effects of BR mediated gain control on awareness-related ERPs and to determine how respiratory dynamics shape perceptual processing independently of direct OB mediated neural entrainment.

**Fig. 1:**
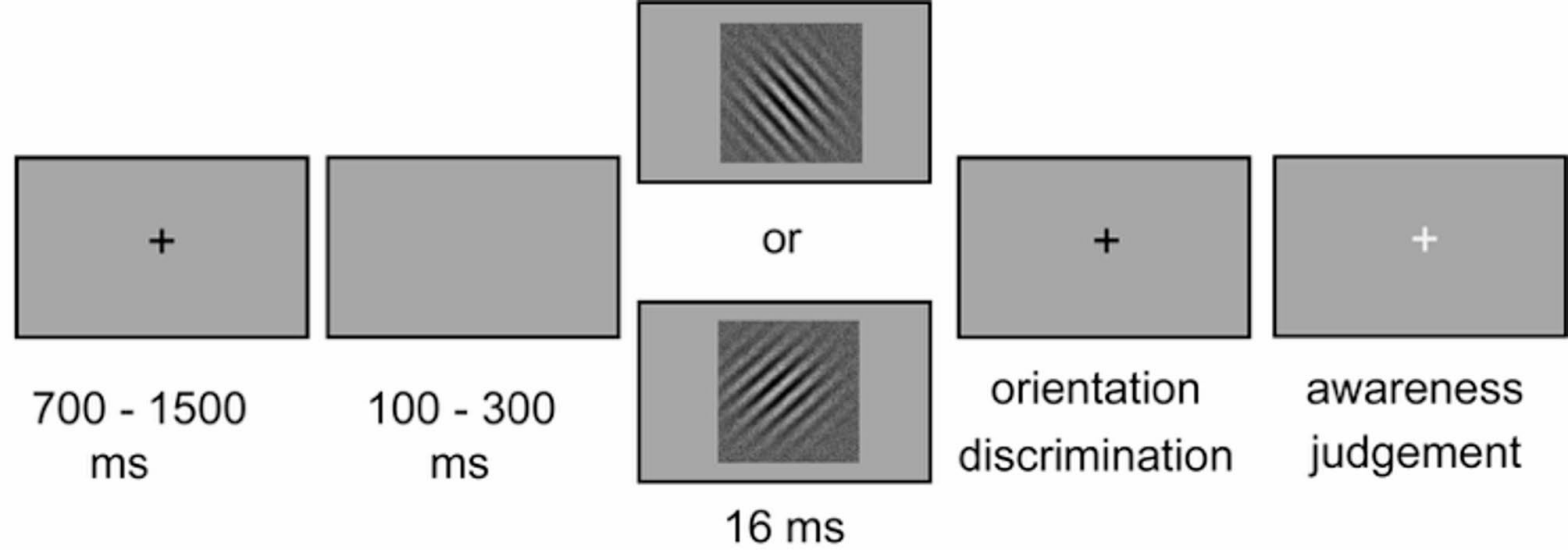
Experimental procedure. A Gabor grating oriented either to the left or to the right was presented for 16 ms. Subjects first indicated the orientation of the stimulus (objective measure of accuracy) and then whether they perceived the stimulus or not (subjective measure of awareness).

To that end, we contrasted ERPs elicited by visual stimuli presented at the discrimination threshold when they were correctly classified with and without awareness (Fig. 1), separately for phases of high (exhalation, systole) and low (inhalation, diastole) BR activity across the respiratory and the cardiac cycles; specifically, we were interested in how awareness-related ERPs differ across bodily cycles when the brain has to process only an exteroceptive stimulus (inhalation, diastole) or simultaneously process both intero- and exteroceptive stimuli (exhalation, systole); we were not interested in the effect of the bodily phase itself on stimulus processing. Subjects had to discriminate between left- and right oriented Gabor gratings embedded in random dot noise and to then indicate whether they saw the stimulus or not (Fig. 1). We equated both performance and physical stimulus properties to avoid confounding awareness and performance. If respiration primarily modulates awareness through indirect BR activity mediated gain control, we would expect early sensory ERP components to be selectively modulated by awareness during inhalation, mirroring the effects seen in the cardiac domain and comparably to nasal respiration. Conversely, if respiratory modulation of awareness relies on OB stimulation, which is absent during oral breathing, any awareness-related ERP differences may shift to later time windows or be less sensitive to the phase.

The cardiac phase was used as a control condition because fluctuations in awareness-related processes during the cardiac cycle are primarily driven by fluctuations in BR activity and should be less affected by the mode of breathing.

## Results

### Behavioral results

**Fig. 2:**
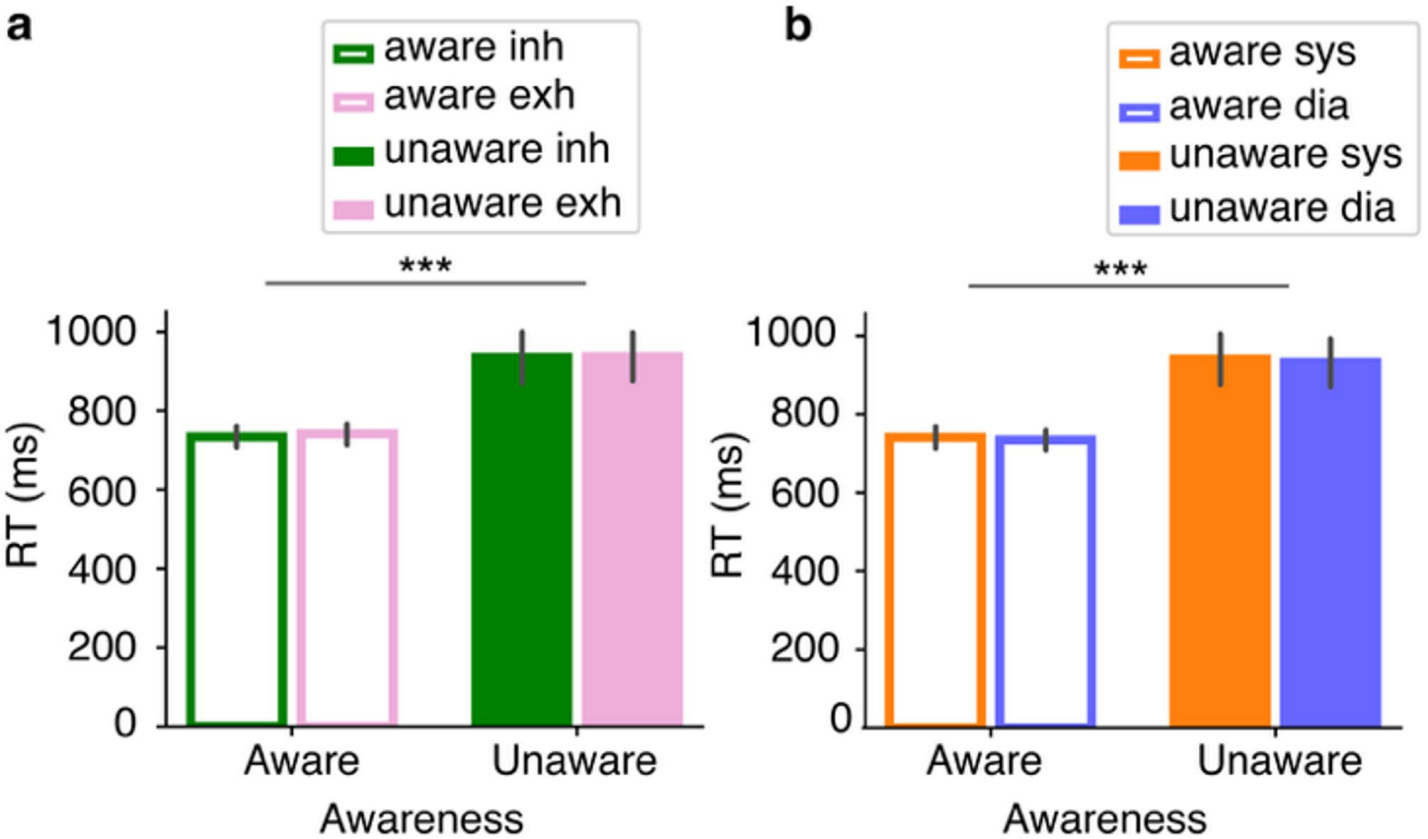
Behavioral results: average reaction times (in ms) as a function of awareness and (**a**) respiratory phase) (inhalation: green, exhalation (pink) and (**b**) cardiac phase (systole: orange, diastole: blue). The error bars represent the standard error, *** *p*<0.001.

Subjects correctly classified stimuli in 87.1% (SD = 6.3%) of trials with a mean ratio of correct aware/unaware responses of 52.6/47.4% (SD = 11.1%), and only correct trials were retained for further analyses. Figure 2 displays the reaction times as a function of awareness and respiratory (Fig. 2**a**) and cardiac (Fig. 2**b**) phase. Reaction times were on average faster in the aware (736 ± 155 ms) than the unaware (935 ± 353 ms) condition. Because RT data were not normally distributed, we applied a Generalized Linear Mixed Model (GLMM) which revealed a main effect of awareness (estimate = 129.28, SE = 3.22, *p* = 10 ^−16^). Neither the respiratory (estimate = − 0.98, SE = 2.92, *p* = 0.74 nor the cardiac (estimate = 1.25, SE = 3.16, *p* = 0.69) phase significantly affected reaction times and neither the respiratory (estimate = − 7.78, SE = 4.02, *p* = 0.053) nor the cardiac phase (estimate = 0.71, SE = 3.92, *p* = 0.86) interacted with awareness.

### Stimulus-evoked potentials

Overall, 13% of trials were discarded due to errors along with 26% of trials falling into the early phase of the diastole and the remainder was removed due to artifacts. An average of 257 trials were kept for aware identification (123/133 in inhalation/exhalation, 128 in both systole and diastole) and 231 for unaware identification (109/122 in inhalation/exhalation, 117/114 in the systole/diastole). The stimulus was only presented for 16 ms, but it elicited clear canonical visual evoked potentials (VEPs).

### Awareness modulates erps

**Fig. 3:**
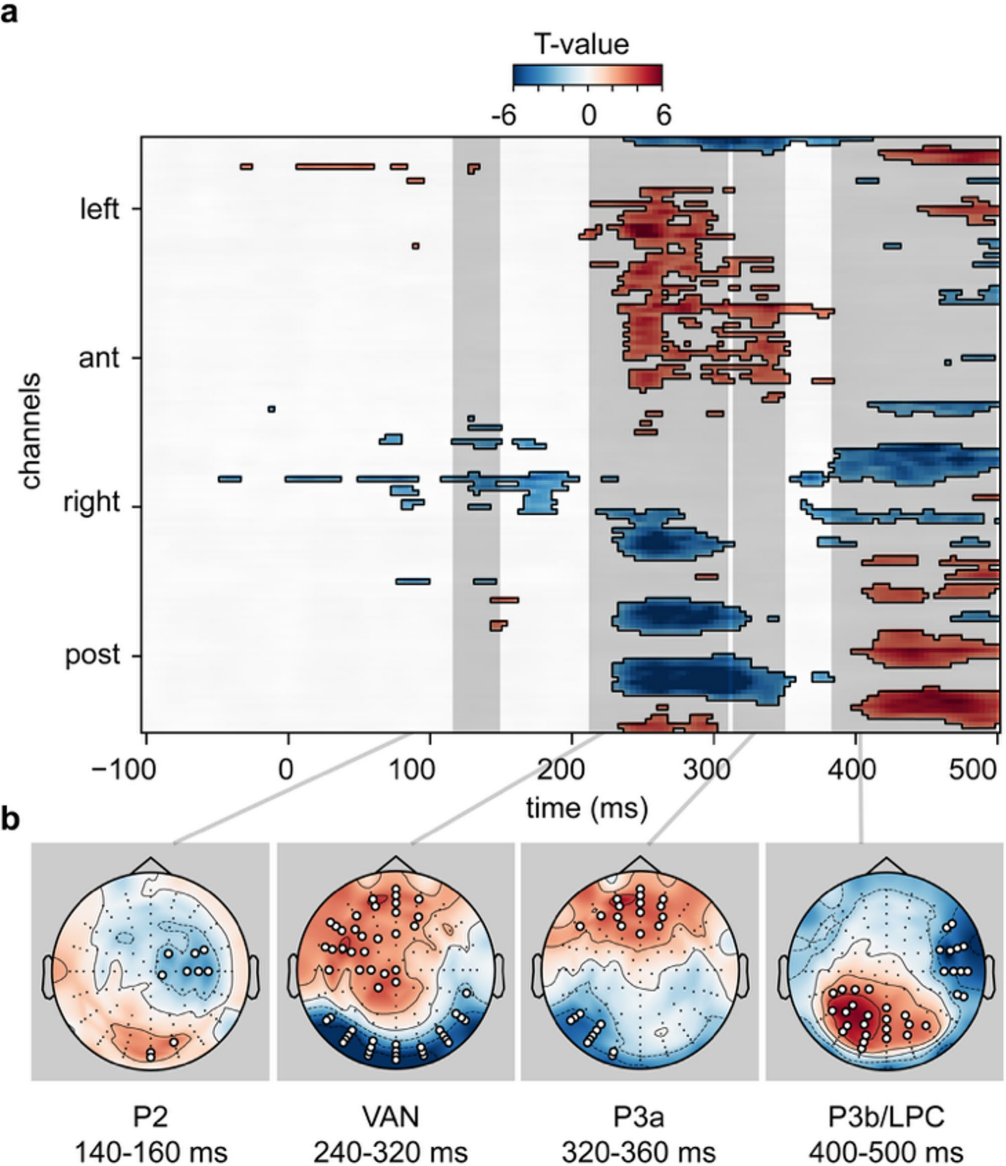
Mass- univariate t-tests contrasting aware and unaware identification of the stimulus (**a**) and corresponding topographic maps (**b**) for the P2 (140–160 ms), VAN (240–320 ms), P3a (320–360 ms) and P3b/LPC (400–500 ms) components. Significant values are FDR corrected (applied over all 128 electrodes and all time-points from − 100–500 ms), blue t-values indicate a negative potential difference and red ones a positive potential difference between correct aware and correct unaware identification; electrodes that showed a significant difference for at least half the time window after FDR correction are indicated by a white dot on the topographic maps.

Figure 3**a** and **b** display four time-windows in which awareness significantly modulated the ERPs (after FDR correction^42^ for multiple comparisons across all 150 time-points and 128 electrodes). First, in the time window between 140 and 160 ms after stimulus onset, ERP amplitudes over posterior electrodes were significantly more positive when the stimulus was identified with awareness compared to when it was identified without awareness. Next, in the period between 240 and 320 ms, the VAN component was significantly more negative for aware identification of the stimulus over posterior electrodes. Next, both the P3a and P3b/LPC components were modulated by awareness: ERPs were significantly more positive over frontal electrodes for the P3a (320 to 360 ms) and over centro-parietal electrodes for the P3b/LPC (400 to 500 ms) for aware compared to the unaware identification.

### Respiratory phase selectively modulates awareness-related erps

**Fig. 4:**
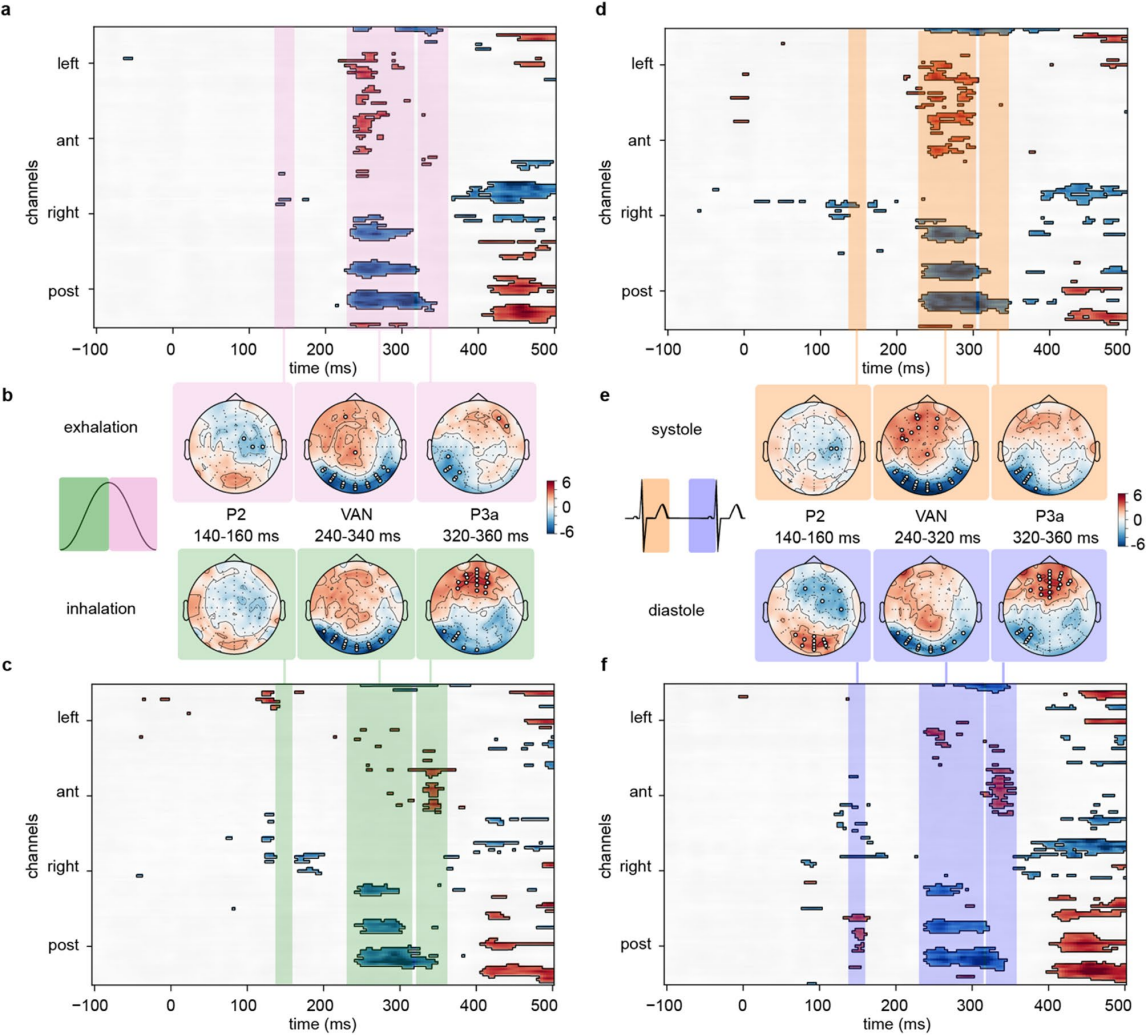
Modulation of the P2, VAN and P3a component*s* as a function of awareness and of the respiratory and cardiac phase. Time course and location of the t -values denoting significant differences between aware and unaware identification of the stimulus *duri*n*g* exhalation (**a**), inhalation (**c**), systole (**d**) and diastole (**f**). The colored boxes highlight the significant period in which the P2 (140-160 ms), VAN (240-320 ms) and P3a (320–360 ms) components *were observed*. **b**, **e**) topo-maps illustrating the distribution of the effects of awareness for exhalation (**b**, top), inhalation (**b**, bottom), systole (**e**, top) and diastole (**e**, bottom). Significant values are FDR corrected (applied over all 128 electrodes and all time-points from −100 to 500 ms), blue t-values indicate a negative potential difference and red ones a positive potential difference between CA and CU *identification*; electrodes *that showed a significant difference* for at least half the time window after FDR correction are indicated by a white dot on the topographic maps.

Figure 4 shows the effects of the respiratory phase on awareness-related ERPs for exhalation (Fig. 4**a**, **b**) and inhalation (Fig. 4**b**, **c**), and Supplementary Fig. 1 shows the grand average ERP waveforms corresponding to the significant differences depicted in Fig. 4 respectively for inhalation (Supplementary Fig. 1**a**) and exhalation (Supplementary Fig. 1**b**). Unlike during nasal respiration^32^, the sensory ERP components were not modulated by awareness, and the VAN was the earliest component modulated by awareness between 240 and 320 ms, and it was equally so during inhalation and exhalation (Fig. 4**a**, **b**, **c**). Unlike for nasal respiration^32^, the P3a component (320–360 ms) was modulated by awareness, albeit only during inhalation, i.e. when the BRs are silent (Fig. 4**b**, **c**) but not during exhalation when they are active (Fig. 4**a**, **b**) with more positive-going ERPs in the aware than unaware identification over fronto-central electrodes. To further assess how the respiratory phase modulated awareness-related ERPs, we compared difference waves (aware – unaware) between respiratory phases (inhalation vs. exhalation). However, none of these comparisons survived FDR correction for multiple comparisons.

### Cardiac phase selectively modulates awareness-related erps

Figure 4 depicts the ERP components modulated by awareness for the systole (Fig. 4**d**, **e**) and diastole (Fig. 4**e**, **f**). Unlike the respiratory phase, the cardiac phase selectively modulated sensory awareness-related ERPs: only in the diastole (silent BRs), but not the systole the ERPs were more positive during the rising phase of the P2 over posterior electrodes in the time-window between 140 and 160 ms when the stimulus was seen than when it was not (Fig. 4**d**, **e**, **f**). Like for the respiratory phase, the P3a was modulated by awareness when BRs were silent during the diastole with more positive-going ERPs over fronto-central electrodes for aware than the unaware identification from 320 to 360 ms (Fig. 4**e**, **f**). During the systole (active BRs), awareness neither affected the P2 nor the P3a components (Fig. 4**d**, **e**). Supplementary Fig. 2 shows the grand average ERP waveforms corresponding to the significant differences depicted in Fig. 4 respectively for the systole (Supplementary Fig. 2**a**) and the diastole (Supplementary Fig. 2**b**). To further examine how the cardiac phase modulated awareness-related ERPs, we compared difference waves (aware – unaware) between cardiac phases (systole vs. diastole). However, none of these comparisons survived FDR correction for multiple comparisons.

## Discussion

In the present study, we investigated how BR-activity mediated gain control across the respiratory and cardiac cycles modulates the neural correlates of perceptual awareness during oral breathing, i.e. in the absence of mechanical OB stimulation. This approach allowed us to clarify the distinct contributions of indirect autonomic influences on awareness mediated via fluctuations of BR activity across the respiratory and cardiac cycles. Our findings demonstrate that both early sensory and later cognitive ERP components associated with awareness are shaped by dynamic interactions with the cardiac and respiratory cycles, but that the underlying mechanisms differ when nasal airflow and OB-mediated neuronal entrainment are absent.

When breathing through the mouth, the early correlates of awareness did not vary with the respiratory phase, i.e. none of the sensory ERP components were modulated by awareness during inhalation or exhalation. These findings clarify the interplay between OB stimulation and BR activity for the conscious processing of a visual stimulus: BR fluctuations alone cannot account for how early sensory processes affect the perceptual outcome. This contrasts with previous results^32^ during nasal breathing where the P1 was the earliest marker of awareness only when OB stimulation was present and BR activity was low. These results suggest that BR-modulated gain control during inhalation requires additional entrainment of brain activity mediated by mechanical stimulation of the OB to affect awareness-related brain activity.

When contrasting aware vs. unaware perception as a function of the cardiac phase, a significant effect of awareness was selectively observed in the diastole during the rising phase of the P2 component, although direct comparison of difference waves did not reach significance after FDR correction. To our knowledge, the P2 has not been shown to be modulated by awareness for visual threshold stimuli. This suggests that even without OB stimulation, BR-mediated influences can still modulate awareness, albeit at a later latency. It also implies that long-term parasympathetic effects during exhalation differ from short-term phasic effects in diastole. Overall, oral breathing abolishes the influence of the respiratory phase on early sensory correlates of consciousness and diminishes the effects of the cardiac phase to a smaller degree.

The subsequent components VAN, P3a, and P3b/LPC components were generally modulated by awareness. Both the VAN and P3b/LPC consistently differentiate between aware and unaware identification in visual discrimination paradigms^2,43^. In contrast, the P3a component was observed only during oral breathing and, to our knowledge, has not previously been reported in ERP studies using sensory discrimination paradigms. It has been reported only once using MEG in an attentional blink paradigm where its amplitude varied with decision confidence^5^. While these effects did not survive direct between-phase statistical contrasts, they suggest a potential sensitivity of later cognitive components to respiratory phase during oral breathing and selectively so for phases of low BR activity (diastole, inhalation).

Both changes in autonomic activity and alterations in oxygen metabolism may explain why the P3a is exclusively modulated by awareness during oral breathing. The P3a is generated in prefrontal cortex (PFC)^44,45^ where oxygen availability is reduced during oral breathing^46^; this depleted state may require greater effort to reach awareness, leading to an increased P3a. Additionally, the P3a is known to vary with novelty^44,47^, task difficulty^48^, habituation^49^ and with the skin conductance response (SCR)^50,51^—the latter being mediated by the sympathetic (SNS) division of the autonomic nervous system (ANS)^52^. In our study, the P3a component was modulated by awareness specifically during diastole and inhalation, when parasympathetic tone is reduced and SNS activity is recruited^40^which ultimately affects the P3a^53^.

The absence of P1 modulation by awareness, coupled with the presence of P3a modulation exclusively during oral breathing indicates that sensory and cognitive processes respond differently to changes in the breathing mode. Future studies could investigate whether this influence of the mode of breathing extends beyond awareness-related brain activity to other sensory and cognitive functions. Longer physiological cycles such as hormonal fluctuations^54,55^ and circadian rhythms^56,57^ also influence autonomic balance and baroreceptor sensitivity. These cycles may interact with respiration and awareness in similar ways and should be explored in future research.

Our analysis focused on evoked (i.e. averaged broad-band / stimulus-induced amplitude changes) rather than induced (frequency-band specific / stimulus-induced frequency changes) EEG activity. This approach allowed us to track the time-course of ERP events with high temporal resolution, which is compromised due to the time-frequency uncertainty principle when investigating induced EEG activity. However, nasal respiration not only entrains band-limited frequency power during tasks^34,38^, but the respiratory phase also differentially modulates low- and high-frequency power in spontaneous resting-state activity^28^. This opens new avenues to investigate whether and how the respiratory phase can modulate task-specific band-limited power like e.g. memory-related theta, attention-related alpha or awareness-related gamma power.

To our knowledge, this is the first time that awareness-related ERPs have been found to vary with the mode of breathing. We show that oral breathing disrupts the BR-modulated gain control influence on early sensory potentials: its effect on the respiratory phases disappears, and its influence on the cardiac phase is delayed. This suggests that to account for differences in gain control, BR activity fluctuations alone are not sufficient, but they require entrainment of brain activity modulated by mechanical OB stimulation. These results align with intracranial recordings in humans^38^ and rodents^35^ which show that oscillatory coordination between brain areas through PAC is present during nasal and absent during oral respiration. This reinforces the view of respiration as an oscillatory scaffold that can modulate brain activity both during oral and nasal breathing^35^. Taken together, our results highlight the importance of recording cardiac and respiratory signals along with brain activity to advance our understanding of the intricate brain-body connection and the interaction between the bodily signals and both the central and peripheral nervous system.

## Methods

### Participants

Thirty-nine healthy right-handed subjects (25 female, age: 24.8 ± 5.1 years, range 18–42) without history of neurological, psychiatric, cardiological and respiratory disorders were recruited for the EEG study. They were the same subjects as in Leupin and Britz^32^and the experimental sessions were two weeks apart with the order counterbalanced between subjects.

The discrimination threshold could not be determined in five subjects and two subjects were excluded from the analysis due to poor signal quality. To maintain consistency in a repeated measure design across both nasal and oral conditions, we further excluded two participants who did not meet the behavioral criteria in the nasal condition. The data of 30 subjects (17 female, age 25.23 ± 5.42 years, range 18–42) was retained for analyses. Participants gave written informed consent and received either monetary compensation (20 CHF/hour) or course credits. The Ethics Committee of the University of Fribourg approved the full study protocol, and the study was conducted in accordance with the Declaration of Helsinki.

### Stimuli and procedure

Target stimuli were Gabor gratings oriented either to the left (135°) or right (45°) and subtending a visual angle of 5° with 3 cpd of visual angle embedded in grayscale random dot noise. Psychopy3 was used to generate and display stimuli on a grey background on a ViewPixx Screen (1920 × 1080 pixel resolution, 120 Hz), and subjects viewed the stimuli in a dimly lit room from a chinrest 70 cm from the screen. Participants were instructed to breathe through their mouth while having a piece of surgical tape placed over the nostrils to refrain from nasal breathing. They performed a threshold determination task followed by the main EEG experiment.

The trial structure was the same for the threshold determination and EEG tasks. A white fixation cross (700 − 500 ms) was followed by a blank screen (100–300 ms) and then by the target stimulus (16 ms). After each trial the subject indicated the orientation of grating by pressing the “F”, (left index) or “J” (right index) key on a keyboard to indicate a left or right orientation, respectively, and then whether they saw (“J”) the stimulus or not (“F”). This measured respectively objective accuracy and subjective awareness of the stimulus.

Before participating in the EEG experiment, each subject had to perform a threshold determination procedure to determine the threshold stimuli that accounted for both performance and subjective awareness. Objective performance and awareness are usually confounded, i.e. aware stimulus identification has to be correct, but subjective awareness is not required to correctly perform in a task^58^. To avoid this confound we kept performance constant and close to ceiling (> 75%) while maintaining similar proportions of correct aware and unaware stimulus identification for each stimulus orientation. Standard adaptive staircase procedures^59^allow the adaptation of only one variable at a time. For this reason, we used a two-step behavioral pre-test in which all stimuli close to the desired range were presented. In the first step, the approximate contrast level was determined by adjusting the Michelson contrast of the random dot noise mask in 20 linear steps from 30 to 100%, following the method implemented by Samaha^60^. In case the difficulty of the task needed to be adjusted, this procedure was repeated by adjusting the opacity of the stimulus. In the second step, we redefined 20 stimuli for each orientation by centering (+/- 20%) the Michelson contrast around the stimuli which yielded values closest to desired threshold. These stimuli were pseudo-randomized and presented for a total 400 trials subdivided in 5 blocks (10 repetitions for each stimulus). This procedure was performed for each stimulus orientation to exclude that awareness was confounded with stimulus orientation, i.e. subjects consistently identified one but not the other orientation.

For the EEG task, we retained the contrast levels that yielded the correct identification for more than 75% of the trials and produced similar identification rates with and without awareness. Participants were presented with a total of 960 stimuli divided into 12 blocks, with each block consisting of 80 trials. To maintain the stability of the threshold, the noise contrast was readjusted throughout the task only if needed.

### Electrophysiological recordings data processing

The EEG was continuously recorded from 128 active Ag/AgCl electrodes (BioSemi^®^) referenced to the CMS-DRL ground. The ECG and respiration were simultaneously recorded along with the EEG at 1024 Hz/16 bit as external bipolar channels. ECG electrodes were placed on the right clavicle and lower left rib, and a respiratory belt (SleepSense^®^) was placed on the lower abdomen.

The cardiac and respiratory signals were preprocessed using the Python Neurokit2 toolbox^61^. The R-peak and the end of the T-wave were marked to indicate the start of systole and diastole. Similarly, the inhalation peak and exhalation trough marked the beginning of the inhalation and exhalation. Trials were then classified according to the cardiac and respiratory phase in which they occurred. We equalized the number of trials across the cardiac cycle to account for the fact that diastole can be twice as long as systole: we only retained trials in which the stimulus fell within the interval at the end of diastole that corresponded to the duration of systole within that cardiac cycle^24,32^, and as a result 26% of the trials were rejected despite fulfilling the desired behavioral criteria. Respiratory cycles that deviated by 2.5 standard deviations faster or 1.5 standard deviations slower than the mean were excluded from further analyses. Only correct trials with (aware) and without awareness (unaware) were included in the analysis.

All EEG analyses, including the pre-processing, averaging, statistical analyses and the creation of images, were performed using the MNE-python toolbox version 1.0.3^62^. The EEG signal was initially re-referenced to the common average reference, filtered between 0.5 and 40 Hz using an FIR filter with a transition window of 10 Hz, and down sampled to 256 Hz. Independent component analysis (ICA) was applied to remove ocular, myogenic and cardiac field artifacts, and trials contaminated by ocular artifacts within 300 ms before and after stimulus onset were rejected. To correct the remaining ocular and myogenic artifacts, the respective ICs were removed, and artifact-free ICs were forward-projected for further analysis. The cleaned data was then segmented into epochs ranging from − 200 ms to 1000 ms around stimulus onset, and artifact rejection and channel interpolation was performed using the Autoreject procedure^63^ implemented in MNE.

### Analysis of behavioral data

Behavioral analysis included only correct responses with aware and unaware identification. Reaction times (RTs) exceeding the 97.5th percentile or falling below the 2.5th percentile were excluded from the analysis. The effects of awareness, and respiratory phase (inhalation/exhalation) and cardiac phase (systole/diastole) on RTs were assessed using two separate General Linear Mixed Effects Models (GLMMs), one for each physiological rhythm. Subjects was included as a random factor in the models, which employed an identity link function with an inverse distribution to account for deviations from the normal distribution typical of RTs^64^. GLMMs were implemented using R Statistical Software (v 4.3.3).

### Analysis of stimulus-evoked potentials

For each subject and for each condition, the epoched data were separately averaged. We contrasted the aware and unaware identification of the stimulus as a function of the respiratory (inhalation, exhalation) and cardiac (systole, diastole) phase. Statistical differences in ERP amplitudes were assessed with mass univariate t-tests in the time window of −100 to 500 ms around stimulus onset. We had no a-priori assumptions about localization or timing of effects and applied False Discovery Rate (FDR)^42^ to compensate for multiple comparisons across time and space.

To assess the effects of cardiac and respiratory phase on awareness-related ERPs, we additionally computed difference waves by subtracting unaware from aware ERPs within each physiological phase for each subject. These obtained waves were then compared across phases (i.e., inhalation vs. exhalation, systole vs. diastole) using paired t-tests. Multiple comparisons across time and electrodes were also controlled using FDR correction.

## Electronic supplementary material

Below is the link to the electronic supplementary material.

Supplementary Material 1

## Data Availability

The code developed during the current study is available in the oral_24 repository78, https://zenodo.org/records/14499167. The consent forms signed by participants do not allow us to give free access to data but require us to check that data are shared with members of the scientific community. Therefore, data are not shared publicly but can be made available upon request to researchers. Please contact the corresponding author Juliane Britz (juliane.britz@unifr.ch).
