# Supplementary material for: Oral respiration modulates sensory and cognitive brain potentials differently than nasal respiration
Source: Sci Rep. 2025 Aug 11;15:29401. doi: 10.1038/s41598-025-12518-1 (PMC12340021; doi:10.1038/s41598-025-12518-1)
Supplement: Supplementary file 1 — Supplementary Material 1 [file 41598_2025_12518_MOESM1_ESM.docx]

**Supporting Information for**

Oral respiration modulates sensory and cognitive brain potentials differently than nasal respiration

## Viviana Leupin^1^ and Juliane Britz^1, *^

## ^1^University of Fribourg, University of Fribourg, Department of Psychology, Rue P.-A. Faucigny 2, CH-1700 Fribourg, Switzerland

## * juliane.britz@unifr.ch

# Supplementary Figures


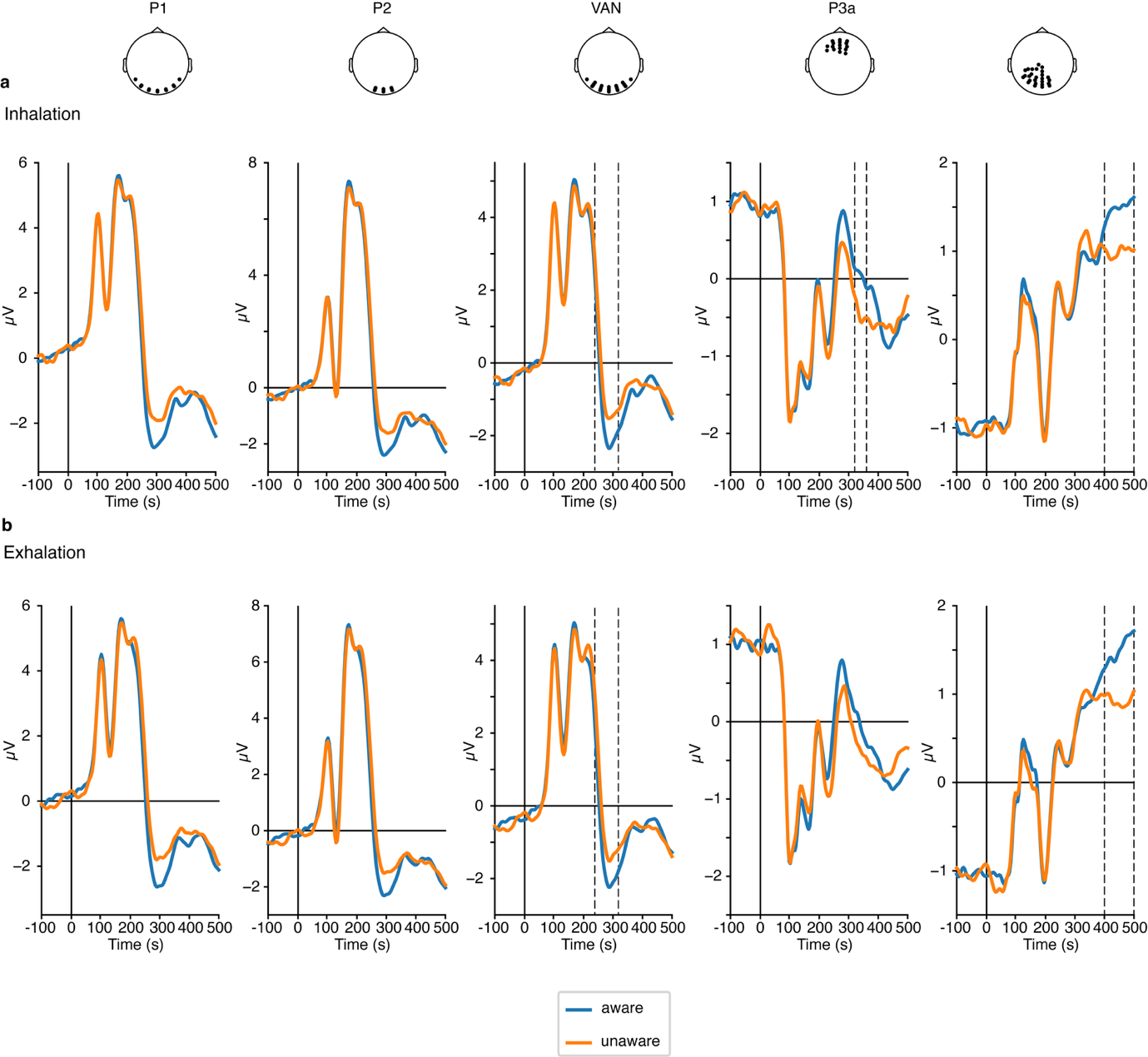


Supplementary Fig. 1. Grand Average ERP waveforms illustrating the significant differences between the aware (blue) and unaware (orange) conditions shown separately for the P1, P2, VAN, P3a and P3b/LPC during **a**) inhalation and **b**) exhalation. The vertical dotted lines indicate time windows where the component was significant in the mass-univariate ANOVAs after FDR correction (see Fig. 4), note that the P1 did not differ between the aware and unaware conditions.


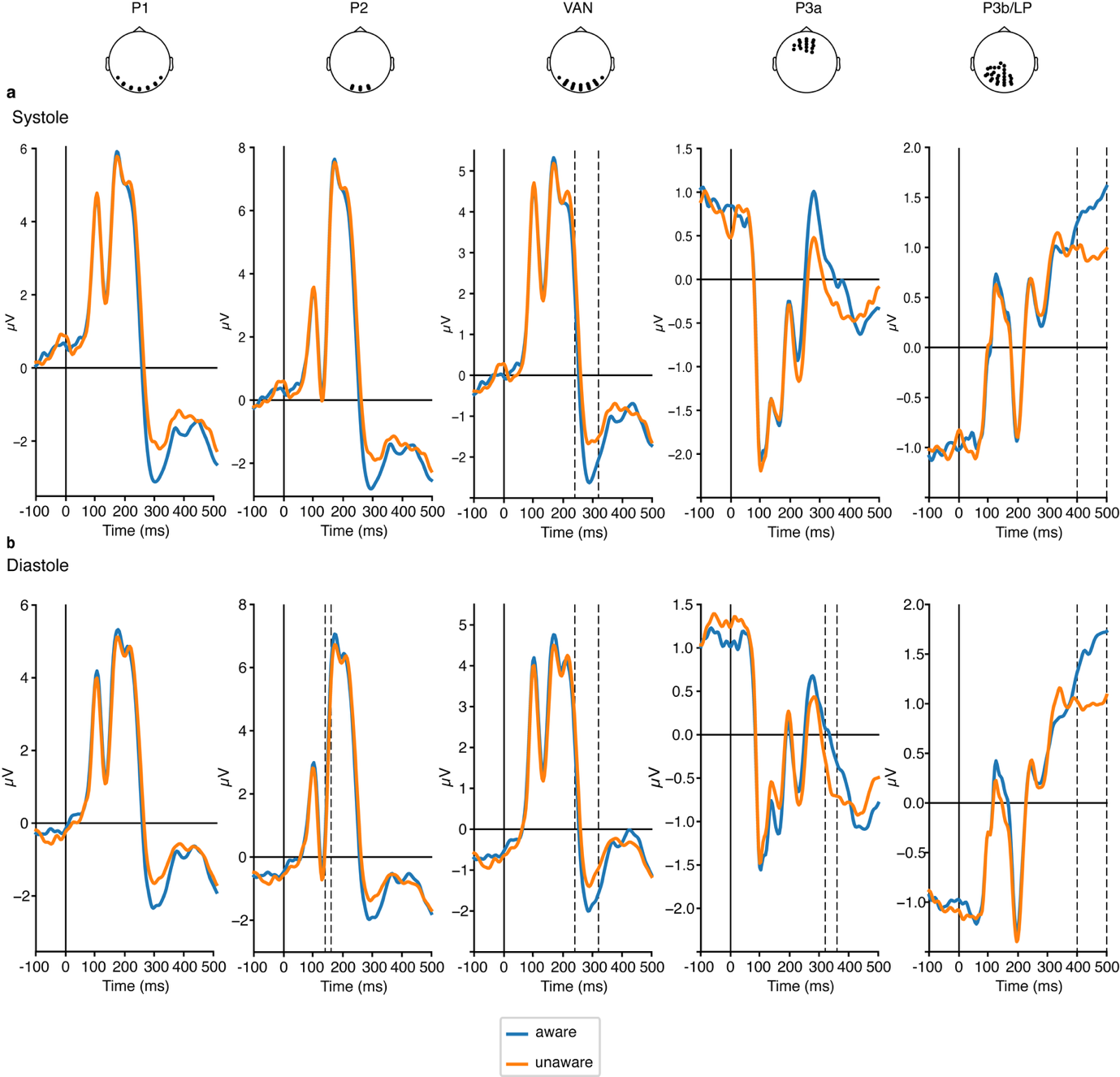


Supplementary Fig. 2. Grand Average ERP waveforms illustrating the significant differences between the aware (blue) and unaware (orange) conditions shown separately for the P1, P2, VAN, P3a and P3b/LPC during **a**) systole and **b**) diastole. The vertical dotted lines indicate time windows where the component was significant in the mass-univariate ANOVAs after FDR correction (see Fig. 4), note that the P1 did not differ between the aware and unaware conditions.
